# A self-powered and self-monitoring ultra-low frequency wave energy harvester for smart ocean ranches

**DOI:** 10.1016/j.isci.2024.110665

**Published:** 2024-08-05

**Authors:** Yang Peng, Hongjie Tang, Hongye Pan, Zutao Zhang, Dabing Luo, Minfeng Tang, Weihua Kong, Yingjie Li, Genshuo Liu, Yongli Hu

**Affiliations:** 1 School of Mechanical Engineering, Southwest Jiaotong University, Chengdu 610031, P.R. China; 2 Chengdu Technological University, Chengdu 611730, China; 3 Yibin Research Institute, Southwest Jiaotong University, Yibin 644000, P.R. China; 4 School of Information Science and Technology, Chengdu 610031, P.R. China; 5 Tangshan Institute of Southwest Jiaotong University, Tangshan 063008, P.R. China; 6 Department of Mechanical Engineering, Beijing Institute of Technology, Beijing 100081, China; 7 Jinan Rail Transit Group Co., Ltd, Jinan, Shandong 250101, China

**Keywords:** Electrochemistry, Electrochemical energy production, Engineering

## Abstract

The ocean ranch environment contains ultra-low-frequency wave energy that can be utilized for powering low-power equipment. Therefore, this article proposes a smart ocean ranch self-powered and self-monitoring system (SOR-SSS) which consists of several key components: a mass pendulum ball (MPB), a commutation wheel system (CWS), an electromagnetic energy harvesting unit (EEHU), and four piezoelectric energy harvesting units (PEHU). Through six-degree-of-freedom vibration test bench experiments, the SOR-SSS achieved a maximum output power of 17.56 mW under a working condition of 0.4 Hz, which was sufficient to power 152 LED lights. Additionally, by training the experimental base data using the LSTM algorithm, two different tasks were trained with a maximum accuracy of 99.72% and 99.80%, respectively. These results indicate that the SOR-SSS holds significant potential for collecting and predicting ultra-low-frequency blue energy. It can provide an effective energy supply and monitoring solution for smart ocean ranch.

## Highlights

• An ultra-low frequency wave energy harvester is designed
• Two gear trains help to improve the working efficiency of the system
• The maximum hybrid output power of 17.56 mW was obtained from SOR-SSS at 0.4 Hz
• Sustainable environmental monitoring was proposed for SOR-SSS based on LSTM models

## Graphical abstract

**Figure:**
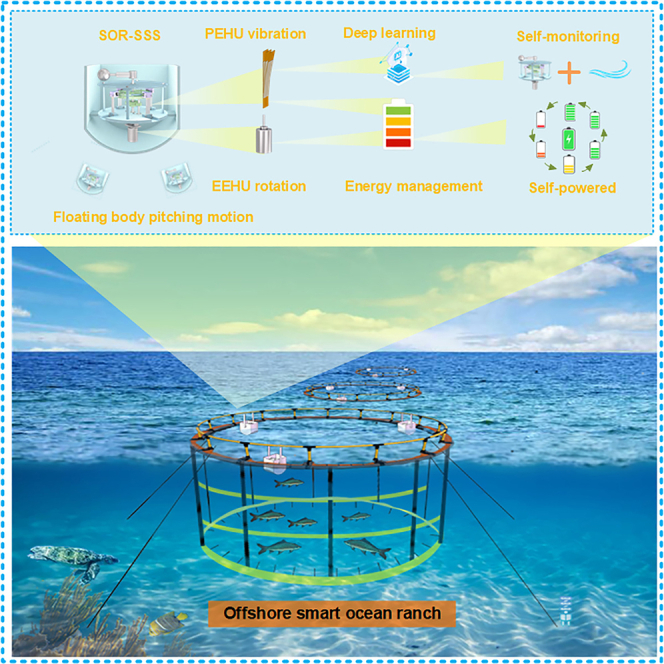

## Introduction

As the world’s population continues to grow and the global climate continues to warm, a new type of ocean ranch called the blue breadbasket has emerged.^1^^,^^2^ These ocean ranches aim to provide high-quality protein resources for the world’s population. Surrounding these ocean ranches are numerous low-power sensors (wireless sensor nodes) that play a crucial role in the real-time collection of ocean environmental data, environmental protection, and monitoring of the ocean’s condition. Traditional sensor nodes, which rely on chemical batteries with limited capacity, short lifespan, and high cost are not sustainable for powering wireless sensor nodes and are being phased out.^3^^,^^4^ Finding alternative energy sources to power these sensors has become a prominent area of research. Blue energy harvesters have shown promise in converting various forms of energy available in the ocean environment, such as wave energy,^5^^,^^6^ solar energy,^7^^,^^8^ and wind energy.^9^^,^^10^ These energy sources are characterized by their instability, irregularity, and low timeliness. However, blue energy harvesters can effectively convert them into regular and efficient electrical energy. Wave energy, in particular, is considered one of the most promising energy sources due to its wide distribution and high energy density.^11^^,^^12^ Harnessing wave energy for the electricity generation offers a viable solution to the challenges posed by traditional chemical battery power supplies, enabling sensors to achieve energy self-sufficiency. Based on existing research, various types of blue energy harvesters have been proposed. From the perspective of the power generation principle of the harvester. These harvesters can be categorized into electromagnetic, piezoelectric, triboelectric, and hybrid systems.^13^^,^^14^^,^^15^^,^^16^

Electromagnetic wave energy harvesters fall into the first category. The principle behind electromagnetic power generation is Faraday’s law of electromagnetic induction.^17^ Li et al.^18^ proposed a low-frequency pendulum-type wave energy capture device that primarily utilizes permanent magnets to create a changing electric field on both sides of the pendulum. This setup allows for the cutting of the coil and subsequent electricity generation. Lou et al.^19^ introduced a biaxial pendulum wave energy collector with excellent adaptability to wave excitation direction. It can capture omni-directional wave excitation, and under experimental conditions of 2.1 Hz, it can produce a peak voltage of up to 14.25 V. Dai et al.^20^ presented a biplane flywheel wave energy harvesting system capable of achieving a peak power output of 201.67 mW at an input excitation frequency of 1.2 Hz. Xie et al.^21^ developed a spatial double-X oscillating float wave energy harvester specifically designed for powering various sensors and monitoring the safety of bridges. The mechanism achieved an average mechanical efficiency of 46.17% in experiments. Li et al.^22^ proposed a range-extending wave-powered autonomous underwater vehicle, demonstrating maximum instantaneous power and average power outputs of 67.74 W and 10.18 W, respectively. This high-performance extended range enables effective expansion of the cruising range of autonomous underwater vehicles.

Piezoelectric wave energy harvesters fall into the second category. Piezoelectric power generation relies on the material’s piezoelectric effect, which converts mechanical energy into electrical energy. Won et al.^23^ introduced a piezoelectric energy harvesting system composed primarily of a piezoelectric cantilever beam structure and a magnet positioned atop the piezoelectric module. He et al.^24^ designed a novel piezoelectric wave energy harvester that utilizes the working mechanism of two floating bodys moving up and down relative to each other. This motion drives the magnet and induces high-frequency vibrations in the piezoelectric sheet, resulting in a maximum output power of 41.5 mW under optimal resistance. Ge et al.^25^ reported on a ball-driven multidirectional ultra-low-frequency piezoelectric vibration energy harvester. At an operating frequency of 0.9 Hz and an external load of 47 kΩ, the device achieved an output power of 41.5 mW. Additionally, by adjusting the external load to 47 kΩ, the output power could reach up to 6.32 mW. Lin et al.^26^ designed a mechanical shock piezoelectric frequency converter and established segmented nonlinear equations of motion using a physical approach. Cai et al.^11^ proposed a small piezoelectric collector based on the acceleration-driven principle. A single piezoelectric sheet in this device can generate a maximum of 5 mW of energy. Shahriar et al.^27^ proposed an energy harvester capable of collecting the pitching and rolling motion of a wave. They primarily investigated the effect of cantilever beam orientation on the root-mean-square voltage in three different directions. Ultimately, through experimentation, they determined that the O_1_ orientation maximized the power density of the device.

Triboelectric wave energy harvesters belong to the third category. Triboelectric power generation relies on the triboelectric effect, which occurs when materials with different properties come into contact, resulting in the transfer of electric charge on the surface of the materials. In 2012, Prof. Zhonglin Wang’s team first proposed a triboelectric nanogenerator based on the triboelectric effect, which effectively converts blue energy into sustained electrical energy.^28^^,^^29^^,^^30^^,^^31^ The stacked triboelectric nanogenerator, proposed by Zhong et al.,^28^ demonstrates a power density more than 13.2 times higher than that of typical spherical-shell structured devices. Han et al.^29^ introduced an uni-directional rotating cylindrical wave drive linkage mechanism based on triboelectric nanogenerators. This mechanism achieves an output power of 50 mW and an output current of 30 μA, enabling it to power devices such as multifunctional barometers. Wu et al.^32^ have made significant progress in the study of triboelectric nanogenerators with continuous output characteristics in the form of alternating current. Zhang et al.^33^ proposed a spherical ID-TENG triboelectric nanogenerator that produces an output current of up to 5 μA at an operating frequency of 30.2 Hz. This output current is 15.2 times higher than that of a device with the same configuration. Li et al.^34^ significantly improved the open-circuit voltage and short-circuit current of the generator by rearranging the electrode arrangement to interleave them.

**Figure 1:**
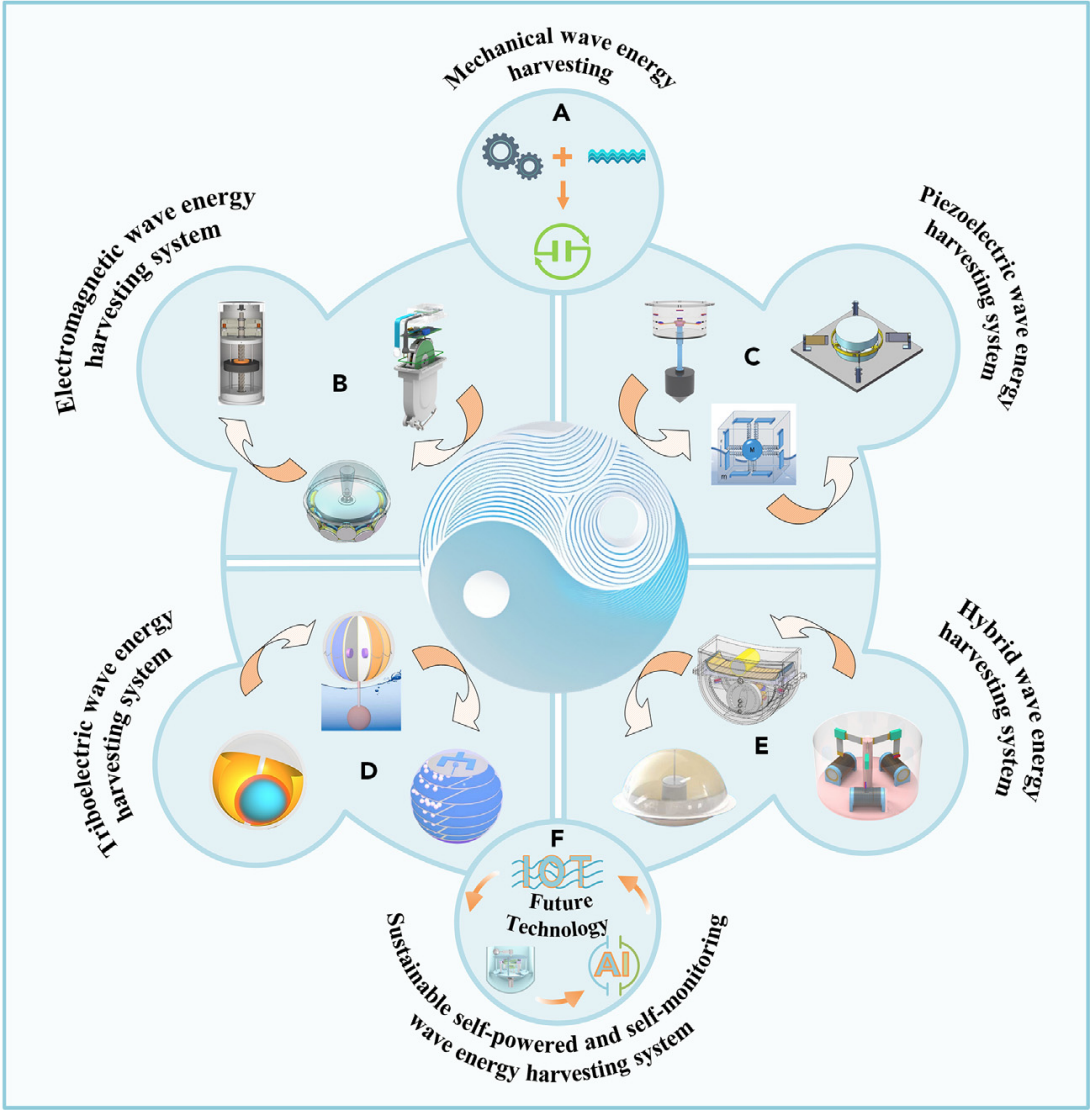
Wave energy harvesting systems with different power generation principles (A) Mechanical wave energy harvesting. (B) Electromagnetic wave energy harvesting system.^18^^,^^19^^,^^20^ (Elsevier Publishing.^18^^,^^19^^,^^20^ Reproduced with permission. All rights reserved.). (C) Piezoelectric wave energy harvesting system.^11^^,^^24^^,^^25^ (Elsevier Publishing.^11^^,^^24^^,^^25^ Reproduced with permission. All rights reserved.). (D) Triboelectric wave energy harvesting system.^30^^,^^31^^,^^33^ (Elsevier Publishing.^30^^,^^31^^,^^33^ Reproduced with permission. All rights reserved.). (E) Hybrid wave energy harvesting system.^39^^,^^41^^,^^42^ (Elsevier Publishing.^15^^,^^39^^,^^42^ Reproduced with permission. All rights reserved.). (F) Future technology.

Many scholars have also proposed hybrid wave energy harvesters based on the principles mentioned above.^15^^,^^35^^,^^36^^,^^37^^,^^38^ Zhao et al.^39^ presented a hybrid harvester that integrates triboelectric, piezoelectricity, and electromagnetism. Experimental results demonstrated that this harvester could continuously power small electronic products. Qi et al.^40^ proposed a capsule-structured electromagnetic-piezoelectric hybrid energy harvester with a maximum output voltage of 7 V and output power of 162 mW. This design holds great potential for powering low-power sensors in cross-sea bridges. The triboelectric hybrid nanogenerator proposed by Ma et al.^41^ is used for efficient hydrogen production in electrolysis tanks, and the system can continuously and stably provide electricity for electrolyzed water, in which the maximum output power of the system can reach 13.8 mW, which makes a strong contribution to the development of carbon neutrality. Zheng et al.^42^ proposed a hybrid wave energy collector based on a magnetic pendulum structure. In a simulated experimental environment, the output voltages and currents of the triboelectric and electromagnetic components reached 90 V, 0.61 μA, and 5.3 V, 6.4 mW, respectively. These results indicate the potential of this collector to self-power sensors for ocean environment monitoring. Tian et al.^43^ developed an energy harvester that combines the three forms of power generation. This hybrid harvester effectively scales up the generation frequency, achieving a maximum power density of 5.73 W/m³. Figure 1 shows different types of mechanical wave energy harvesting systems from the perspective of each of the four power generation principles, and concludes with an outlook on the future development of sustainable self-supplying and self-supervised wave energy harvesting systems in the context of existing technologies.

With the increasing research focus on wave energy harvesting in recent years. Depending on the functions they can achieve, wave energy harvesters can be classified into three types: self-driven, self-monitoring, and hybrid combining both functionalities. Many of the blue energy harvesters mentioned earlier can be categorized as self-driven types based on their power generation principles. Hybrid types, which combine self-driven and self-monitoring functionalities, have been widely employed in the field of human energy harvesting. However, research on blue energy harvesters that integrate both self-driven and self-monitoring functions is still in its early stages. It has been shown^44^^,^^45^ that a Piezoelectric transducer has high sensitivity and wide frequency response characteristics, an electromagnetic transducer has better power generation performance, and the combination of the two transducer modes, in charging the capacitor than the two individual transducer modes has more obvious advantages, on the one hand, PEHU can solve the problem of providing a higher voltage, on the other hand, EEHU can solve the problem of providing a larger charging current. Therefore, this article proposes a self-powered, self-monitoring system for smart ocean ranch. In this system, a response bearing is attached to the free end of the PEHU, while a cam is attached to the edge of the large gear wheel in the commutation gear train. Increasing the number of cams can enhance the operating frequency of the piezoelectric transducer, thereby increasing the output power per unit of time. Excited by the ocean waves surrounding the ocean ranch environment, the floating body generates pitching motion, and the SOR-SSS inside the floating body generates relative motion. These motions cause the MPB to undergo random bidirectional rotation. The CWS converts these bidirectional rotations into electrical signals generated by the EEHU and the PEHU. The electrical signals generated by the EEHU enable self-power supply. The electrical signals generated by the PEHU are used in two ways. On one hand, they are used to achieve self-powering functionality, and on the other hand, they will be integrated with the LSTM algorithm to realize self-monitoring of the surrounding environment of the ocean ranch and the working status of the SOR-SSS system. The rest of the important chapters of this article are organized into four aspects: system design and principle, system dynamic analysis, test and result discussion, and conclusion.

### System design and principles

#### System design

**Figure 2:**
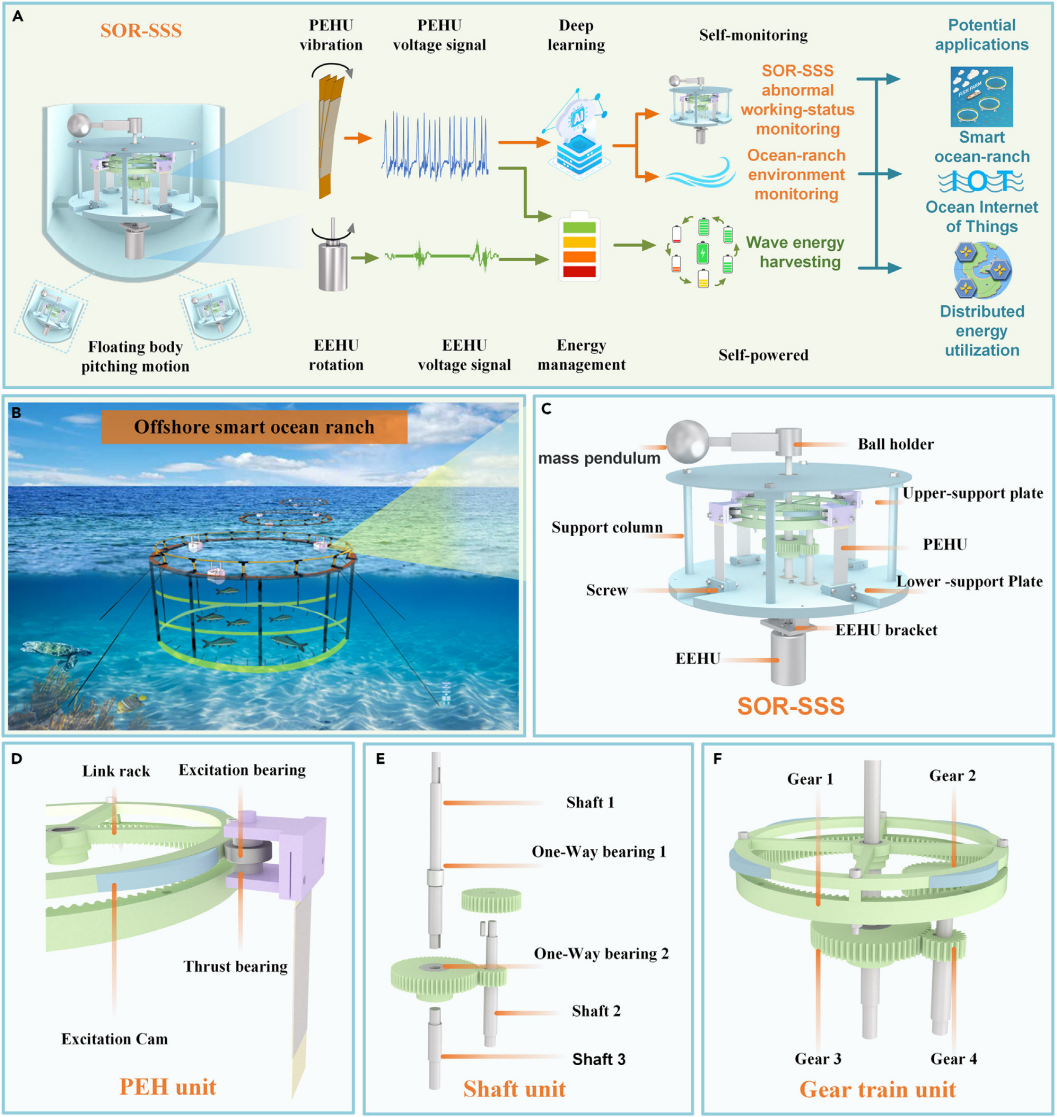
Schematic diagram of the SOR-SSS (A) The proposed workflow diagram of the SOR-SSS. (B) The application of the SOR-SSS in the offshore ocean ranch. (C) The key construction name diagram of the SOR-SSS. (D) The piezoelectric excitation unit of the SOR-SSS. (E) The shaft unit of the SOR-SSS. (F) The gear train unit of the SOR-SSS.

Figure 2A illustrates the system workflow of SOR-SSS. The system is designed to be installed inside a floating body that will do pitching motion. When the floating body is stimulated by waves, it generates a pitching motion, which drives the SOR-SSS inside the floating body to generate electricity. The electrical signal generated by the EEHU enables the self-power supply function. Due to the piezoelectric effect, the electrical energy generated by the stimulated PEHU is used to supply power to low-power sensors in the surrounding environment. Additionally, it is used to monitor the system’s stability and the wave height in the ocean environment using the LSTM algorithm to analyze the electrical signal. SOR-SSS serves as an effective network node in offshore smart ocean ranch internet of things systems (IOTs). It generates power to supply other devices and provides feedback on the ocean conditions, contributing to the high-speed and sustainable development of offshore ocean ranch IOTs. The floating bodys with SOR-SSS are arranged equidistantly on racks of fishery aquaculture nets, which are fixed evenly on the seabed with four mooring lines, as depicted in Figure 2B. The main structure of SOR-SSS is shown in Figure 2C. In addition to standard parts such as screws, nuts, and bearings, the system consists of the main units: MPB, CWS, PEHU, and EEHU. The local details of SOR-SSS are enlarged in Figures 2D–2F. Figure 2D displays the PEHU, which shows the cam on the edge of the link frame about to contact the excitation bearing. Figure 2E depicts the shaft unit of the system, highlighting Shaft 1, Shaft 2, Shaft 3, and two thin-walled One-Way bearings. The thin-walled One-Way bearings work in tandem with the gear unit, collecting the kinetic energy generated from the randomized bidirectional rotation of the MPBs. Figure 2F showcases the gear train unit of SOR-SSS, comprising two pairs of gears: Gear 1 with Gear 2 and Gear 3 with Gear 4.

#### The principle of variable frequency excitation of the system

The piezoelectric frequency enhancement technology based on physical contact has emerged as a research focus in recent years.^46^^,^^47^^,^^48^ The average frequency of offshore wave occurrences is approximately 0.2 Hz.^49^ Considering the actual ocean conditions in offshore ocean ranchs, this article aims to improve the working efficiency of the PEHU in SOR-SSS. To achieve this, we employ a variable frequency excitation mechanism and install a cantilever beam bicrystalline piezoelectric sheet around the cam. The main parameters of the piezoelectric sheet are presented in Table 1. To reduce friction, thrust ball bearings are installed at the upper and lower ends of the excitation bearing. Additionally, different numbers of cams with varying projection distances are added to gear 2. When the MPB rotates randomly, the cams on the edges of the connecting frame immediately acquire kinetic energy. The excitation bearing slides along the edges of the connecting frame when the PEHU is not excited. However, when the cams contact the excitation bearings, the cantilever beam bicrystalline piezoelectric sheet is deformed, resulting in the generation of an electrical signal. Since the system operates under different ocean conditions, the piezoelectric signal exhibits high voltage and high sensitivity to the environment, making it suitable for accurately identifying real-time ocean conditions and the system’s operating conditions. By processing the electrical signal of the PEHU after frequency conversion using algorithms, an effective method for self-monitoring can be established.

**Table 1:** Main parameters of cantilever beam bicrystalline piezoelectric sheet

Parameter	Value
Base length	100 mm
Base width	20 mm
Base thickness	0.2 mm
Lengh of piezoelectric material	60 mm
Width of piezoelectric material	20 mm
Thickness of piezoelectric material	0.2 mm
The density of the base layer	8.78 × 10^3^ kg×m^−3^
The density of the piezoelectric layer	7.50 × 10^3^ kg×m^−3^
relative dielectric constant	3400
Young’s modulus	56
Poisson’s ratio	0.36

#### The principle of the commutation rectification of the system

**Figure 3:**
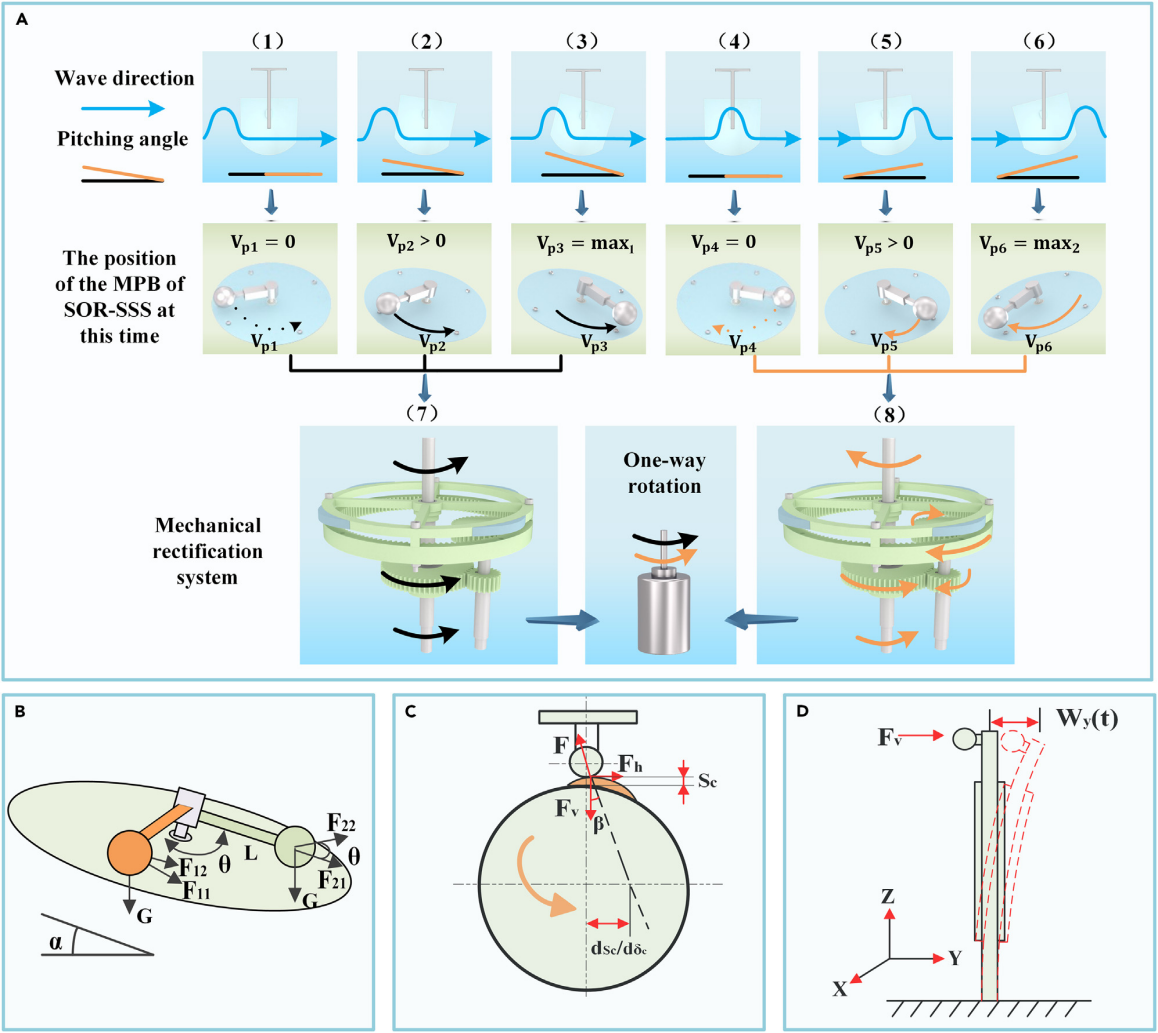
Detailed drawing of SOR-SSS movement principle (A) The motion state of the SOR-SSS inside the floating body under the wave excitation in one period. (B) schematic diagram of the top force of the SOR-SSS. top view of the SOR-SSS. (C) Stress analysis diagram of cam. (D) Equivalent piezoelectric patch to cantilever a beam.

The commutation rectifier unit is a common component in mechanical design. Its purpose is to convert the input module with bi-directional motion into the output module through the commutation rectifier unit, enabling the output module to have a single direction, thereby improving system efficiency.^50^^,^^51^ Given the indeterminate rotation of the MPB, we have designed a commutation rectifier unit specifically for the SOR-SSS. When the MPB rotates clockwise and drives shaft 1 in the same direction, gear 1 meshes with gear 2. As a result, gear 2 drives gear 4, which is on the same shaft 2, to rotate. Gear 4 then meshes with gear 3, which subsequently drives shaft 3 to rotate (The detailed motiom principle is shown in Figure 3A). Similarly, when the MPB rotates counterclockwise, the thin-walled One-Way bearings allow the MPB to directly drive shaft 1 and shaft 3 to rotate counterclockwise, thereby driving the motor rotation. Through this process, regardless of the direction of the MPB’s rotation, the commutation gear system ensures that the output shaft can only output in a single direction. Consequently, the EEHU operates in a unidirectional manner. As gear 2 rotates uni-directionally under the influence of the two thin-walled One-Way bearings, The cam of the connecting frame 2 is continuously excited by the PEHU in one direction, so that the hybrid power generation is realized.

### System dynamic analysis

**Figure 4:**
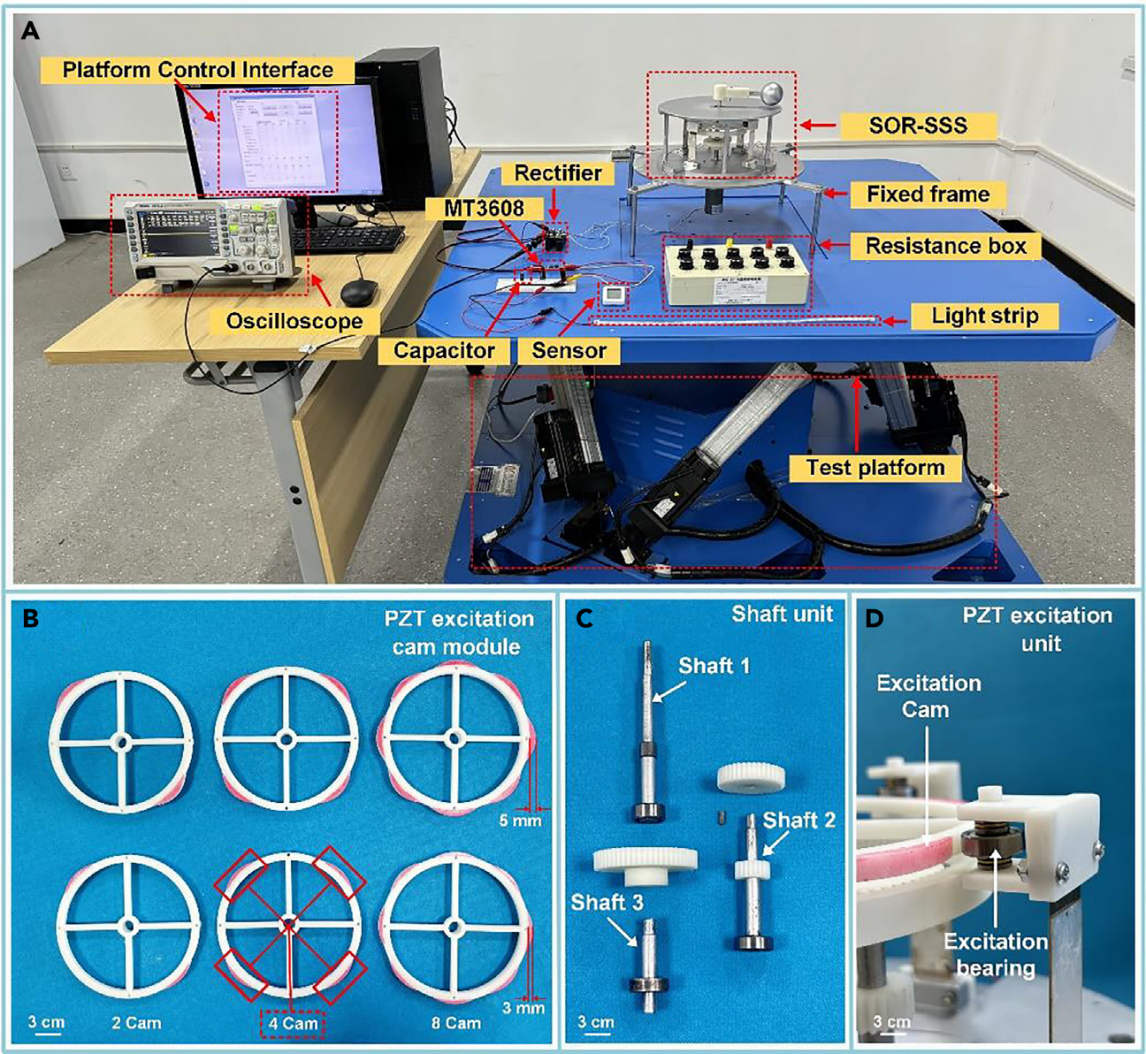
Experimental bench and detail display (A) Six-degree-of-freedom vibration test bench supporting experimental equipment display. (B) Piezoelectric excitation cam module. (C) Shafting unit. (D) Piezoelectric excitation module.

**Figure 5:**
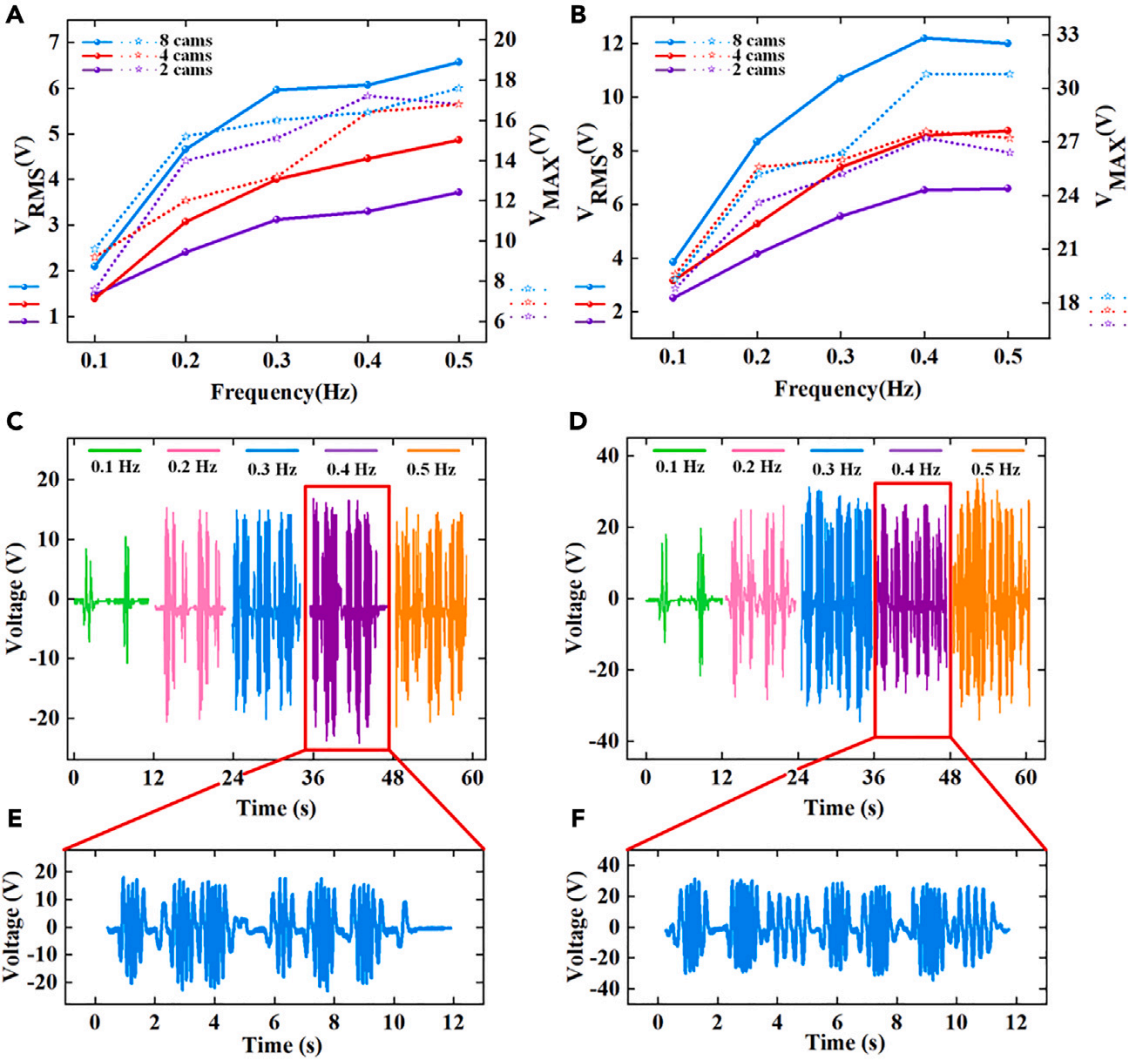
System performance diagram under different cam numbers (A) D = 3 mm electrical signal of PEHU. (B) D = 5 mm electrical signal of PEHU. (C) D = 3 mm electrical signal of PEHU at each frequency for the number of 8 cams. (D) D = 5 mm electrical signal of PEHU at each frequency for the number of 8 cams. (E) D = 3 mm electrical signal of PEHU at 0.4 Hz for the number of 8 cams for the 12s. (F) D = 5 mm electrical signal of PEHU at 0.4 Hz for the number of 8 cams for the 12s.

**Figure 6:**
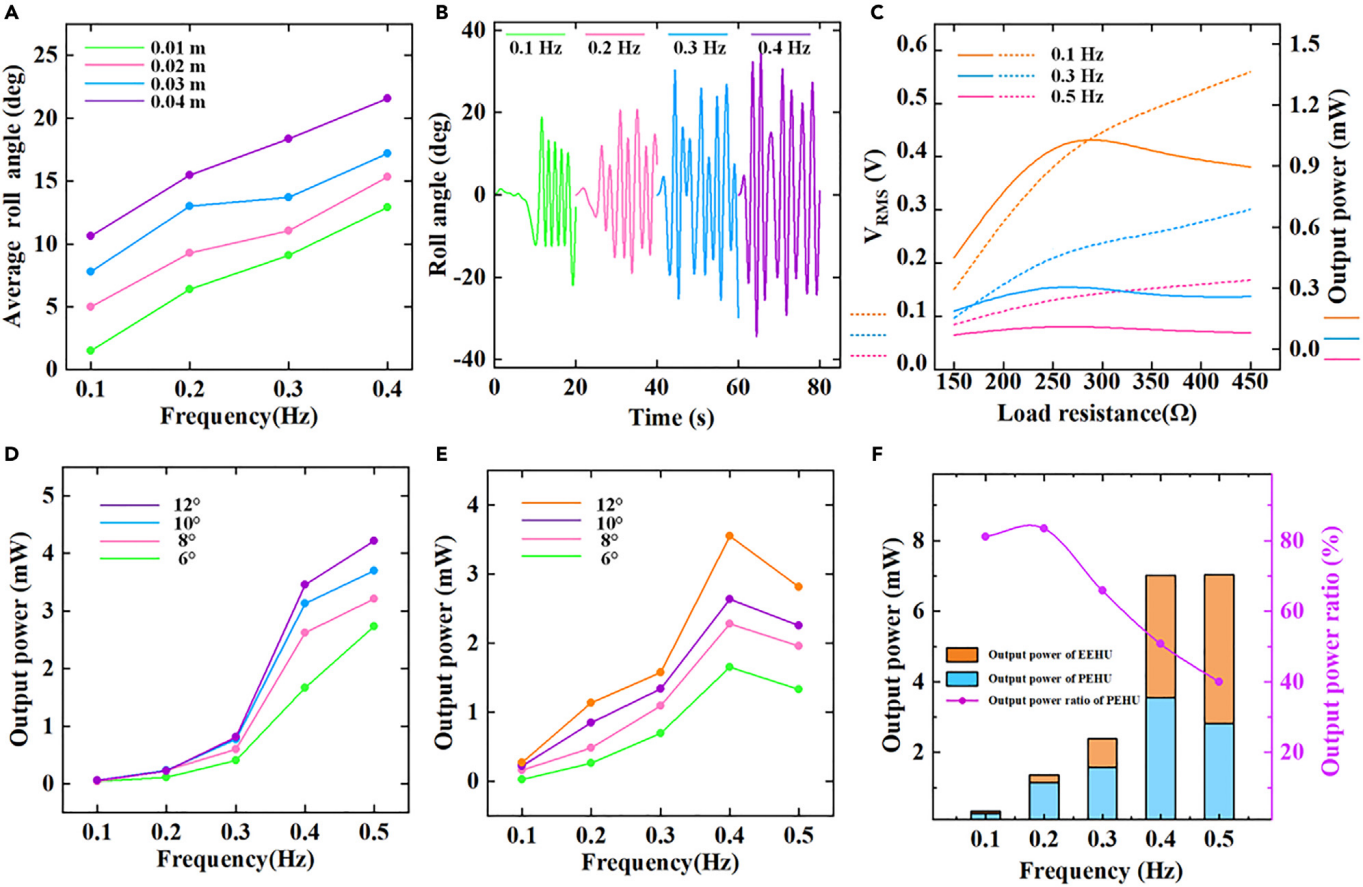
System power generation performance diagram under different operating conditions (A) Average response longitudinal rocking angle of the floating body at different wave amplitudes and frequencies in ANSYS AWQA. (B) Time domain longitudinal rocking angle of the floating body at different frequencies with wave amplitude at 0.04m. (C) Optimal load profile of PEHU. (D) Output performance of PEHU at different frequencies. (E) Output performance of EEHU at different frequencies. (F) Comparison of output performances of PEHU and EEHU at different Comparison of output performance at different frequencies.

**Figure 7:**
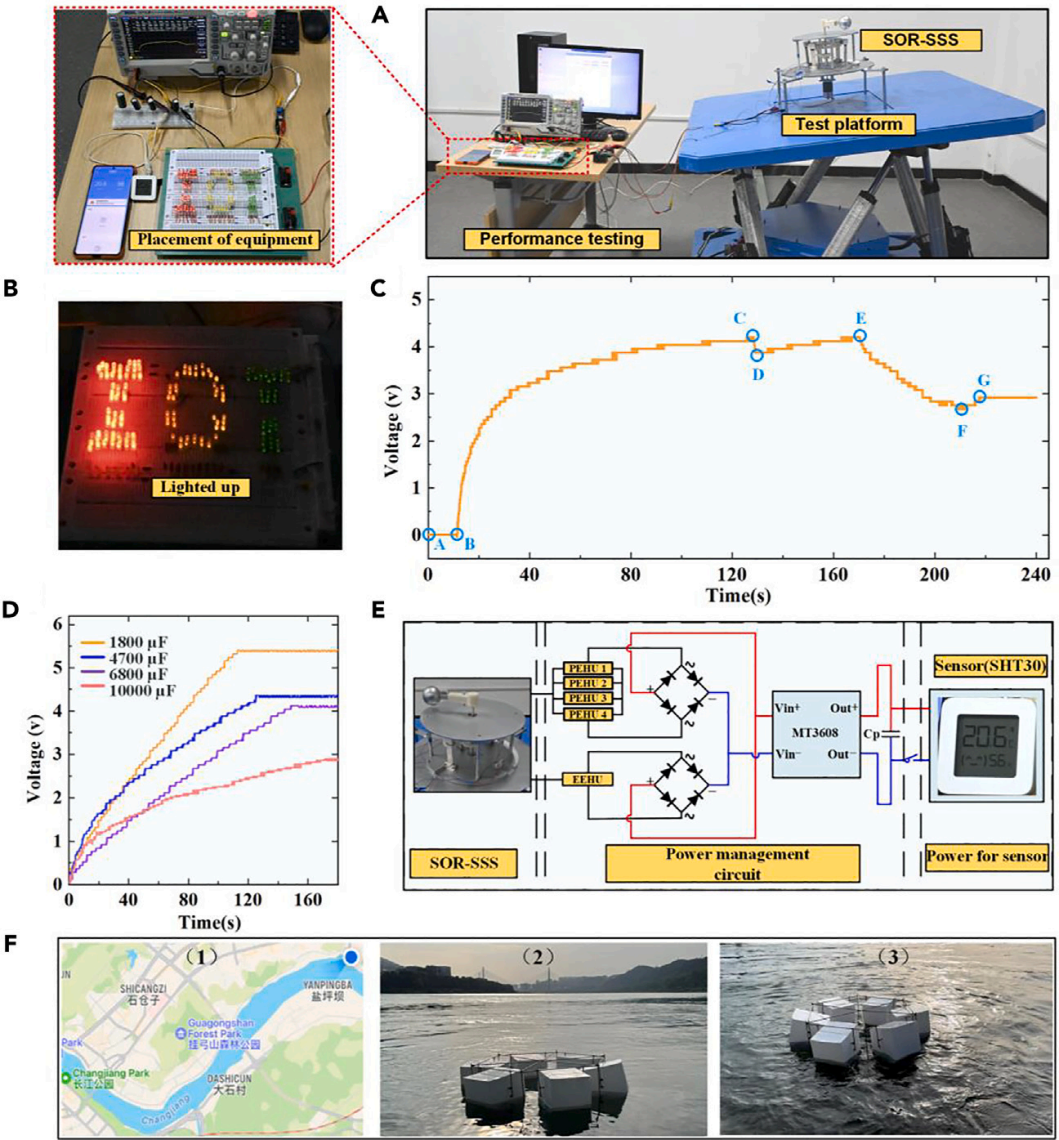
Description of other experiments (A) Charge and discharge experimental environment. (B) Lights up 152 LEDs. (C) Hybrid charging of four different capacitors. (D) Operating curves of 6800 μf capacitors. (E) Power supply principle circuit. (F) Field verification floating body pitching test in the Yangtze River.

**Figure 8:**
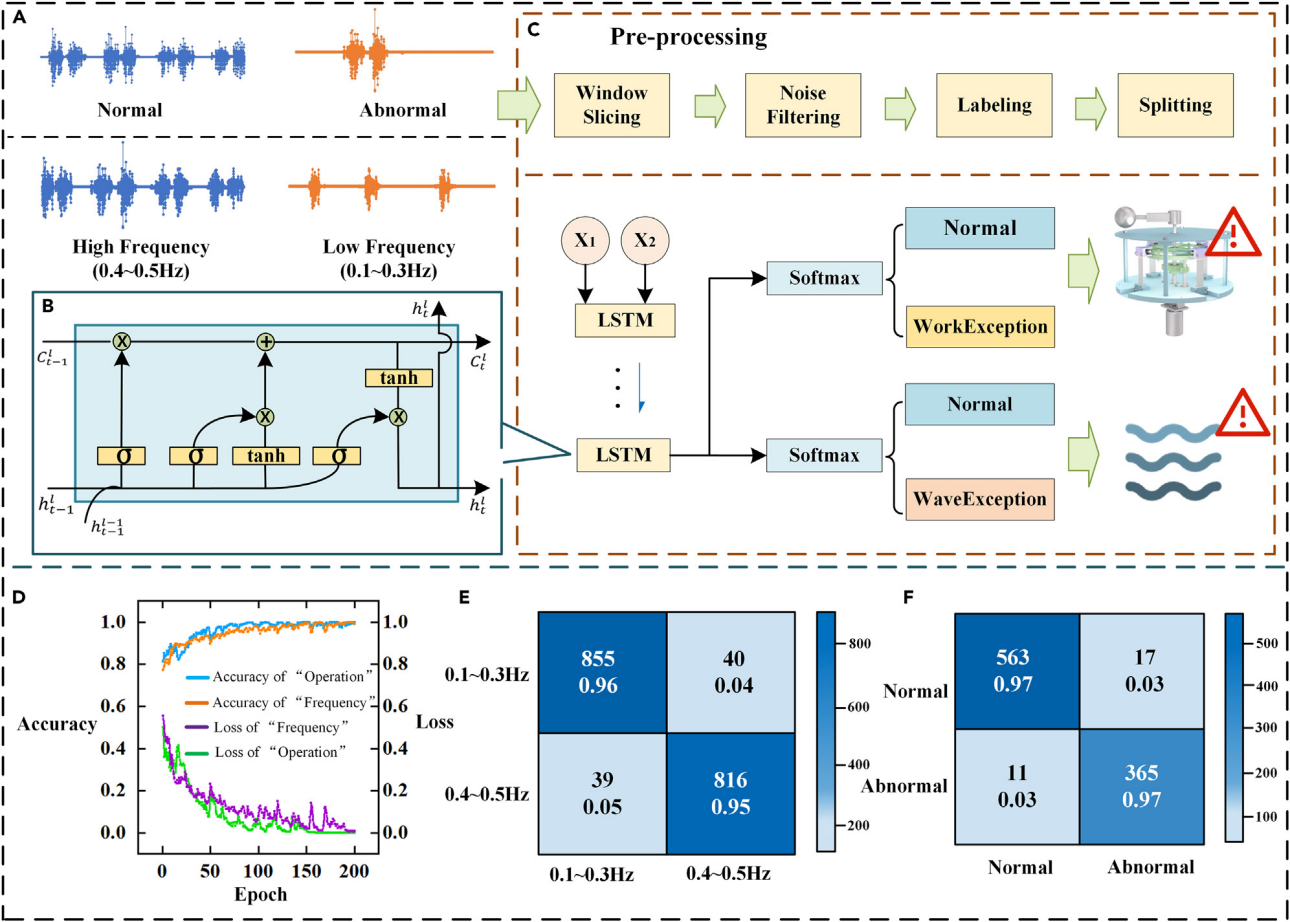
Flow chart of smart ocean ranch monitoring based on LSTM (A) Origin data input collected in different condition. (B) Structure of LSTM model. (C) Data processing and training. (D) Training accuracy and loss of classification tasks. (E) Confusion matrix of frequency classification. (F) Confusion matrix of operation condition classification.

The proposed SOR-SSS in this article is designed to be installed inside a floating body. The floating body is mounted on a pedestal through a hinge, and when it is excited by waves, it will do a pitching motion. Figure 3A shows the six different motion states presented by the floating body of SOR-SSS under the wave excitation in the unit cycle and its corresponding working state of SOR-SSS, the wave propagates from left to right, as shown in Figure 3A -(1) the SOR-SSS is in dynamic equilibrium, and no relative motion occurs at this time the velocity of the MPB is 0 (V_P1_ = 0) and as the crest of the wave continues to move to the right, the Horizontal plane and the MPB movement plane angle (pitch angle) is increasing, in Figure 3A-(3) pitch angle to reach the maximum value of the inclination to the right, at this time the speed of the MPB has also reached the maximum value of the moment of the right inclination (V_P3_ = MAX_1_), similarly, with the continued propagation of the wave, the SOR-SSS in Figure 3A-(6) pitch angle to reach the maximum value of the inclination to the left, at this time the speed of the MPB also reaches the maximum value at the moment of rightward inclination (V_P6_ = MAX_2_), From Figure 3A-(1) to Figure 3A-(3)and Figure 3A-(4) to Figure 3A-(6),The work flow of the CWS inside the SOR-SSS is shown in Figure. Figure 3A-(7) and 3A-(8), and although there are different rotational directions of the MPB at different pitch angles, it finally drives the PEHU to rotate unidirectionally due to the rectification effect of the CWS. Since the SOR-SSS is fixed inside the floating body, the inclination angle of the floating body with respect to the wave surface is the same as the angle of inclination within the SOR-SSS with respect to the wave surface, denoted as α. Assuming that the waves in the ocean ranch environment behave as an ideal fluid, the equation of the incident wave surface at the sea surface can be described as follows:

(Equation 1) $$\mu \left(x，t\right)=\frac{H}{2}\sin\left(nx-wt\right)$$

In Equation 1, μ represents the wave surface, H denotes the wave height, n is the number of incident waves, and w represents the wave frequency of the incident waves. The pitching angle of the floating body is approximately equal to the inclined slant angle of the wave at time t. To simplify the theoretical model, we record the inclined angle of the wave at time t as α when x = 0.

(Equation 2) $$\alpha =\arctan\;\left(\frac{-Hw}{2}\cos\left(wt\right)\right)$$

The MPB in Figure 3A-(2) and 3A-(3) position force diagram simplified in Figure 3B shown, due to the process of gravity force is much greater than the mechanical part of the resistance, so the analysis of the process of the force, ignoring the mechanical part of the resistance, assuming that the ideal state, and therefore by the gravity force generated by the moment of the equation can be obtained.

(Equation 3) $$MgR\;\sin\;\theta \;\cos\;\alpha =I\dot{\gamma }$$

where M is the mass of the MPB, θ is the angle at which the MPB is turned, α is the angle of inclination of the floating body in Equation 2, and $\dot{\;\gamma \;}$ is the angular acceleration.

(Equation 4) $$I=MR^{2}$$

where R is the radius of rotation of the MPB, which can be obtained by bringing Equation 4 into Equation 3:

(Equation 5) $$\dot{\gamma }=\frac{g\;\sin\;\theta \;\cos\;\alpha }{R}$$

As depicted in Figure 3D, the piezoelectric beam is excited in the Y-direction. Taking into account the electro-mechanics of the piezoelectric beam and considering the complexity and diversity of its vibration model, this article focuses on the first-order bending mode of vibration of the piezoelectric beam. The control equations governing its response state are presented as follows.^52^

(Equation 6) $$M_{P}{\ddot{W}}_{y}+\eta_{p}{\dot{W}}_{y}+K_{P}W_{y}+\delta V_{P}=F_{V}$$

(Equation 7) $$-\delta {\dot{W}}_{y}+C_{P}{\dot{V}}_{P}=-I_{P}$$

where $M_{P}$ is the effective mass of the piezoelectric beam with first-order bending vibration mode, W_y_ is the displacement of the piezoelectric beam in the direction of excitation, $\eta_{p}$ is its damping coefficient, $K_{P}$ is the effective stiffness of the piezoelectric beam, δ is its effective piezoelectricity coefficient, $C_{P}$ is the clamping capacitance of the piezoelectricity, and $V_{P}$ and $I_{P}$ are the voltage and current of the voltage output, respectively.

As shown in Figure 3C, the cam and the piezoelectric part are collisional excitations, which are obtained from the momentum theorem.

(Equation 8) $$\bar{F_{V}}\Delta t=m\Delta v$$

(Equation 9) $$\Delta v=v_{1}-v_{0}$$

where ${\;F}_{V}$ denotes the force on the piezoelectric beam in the Y-direction, Δt is the time difference during the collision, m is the mass of the piezoelectric beam, and Δv is the velocity of the piezoelectric beam in the Y-direction before and after the collision. Here the starting velocity $v_{0}=0$. Since the cam and piezoelectric excitation parts do not separate after the collision, according to the three-center theorem.

(Equation 10) $$v_{1}=R_{1}\gamma \;\cos\;\beta$$

(Equation 11) $$\beta =\arctan\left(\frac{d_{S_{C}}/d_{\delta_{c}}}{S_{C}+r_{0}}\right)$$

Equation 10 where R_1_ denotes the distance from the collision position to the center of rotation of the toothed ring 1, and β is the pressure angle of the cam. Equation 11 where $r_{0}$ denotes the radius of the cam base circle and $S_{c}$ denotes the lift. Since the above MPB and toothed-ring commutation unit are regarded as a whole, i.e., the acceleration of the MPB is equal to the acceleration of the toothed-ring 1, the angular velocity of the toothed-ring 1 can be obtained from the Equation 5:

(Equation 12) $$\gamma =\int_{t_{1}}^{t_{2}}\frac{g\;\sin\;\theta \;\cos\;\alpha }{R}dt$$

The main parameters of the motor selected in this article are shown in Table 2, in the process of motor power generation, the magnitude of the amplitude voltage is in the case of a certain motor reverse electromotive force constant with the speed of the motor rotor shaft changes, which can be expressed as:

(Equation 13) $$E_{m}=K_{e}\gamma$$

where $K_{e}$ is the reverse electromotive force constant and γ the rotation speed of the motor. The three AC brushless motors selected in this article as power generators produce voltages at each stator which can be expressed as:

(Equation 14) $$U_{1}=E_{m}\;\sin\left(w_{e}t-120{}^{\circ}\right)$$

(Equation 15) $$U_{2}=E_{m}\;\sin\left(w_{e}t\right)$$

(Equation 16) $$U_{3}=E_{m}\;\sin\left(w_{e}t+120{}^{\circ}\right)$$

where $U_{1}$, $U_{2}$ and $U_{3}$ denote the induced voltage of each stator coil, where $E_{m}$ and $w_{e}$ denote the amplitude and angular frequency of the voltage, respectively. The total instantaneous power $P_{E}$ of the EEHU is equal to the sum of the power captured in the circuit $P_{e}$ and the power lost in the circuit $P_{l}$, which can be expressed as：

(Equation 17) $$P_{E}=P_{e}+P_{l}$$

**Table 2:** Main parameters of the motor

Parameters	Value
Rated voltage	230 V
Rated current	0.09 A
Rated power	17 W
Rated torque	0.08 Nm
Rated speed	1400 rpm
Rotor inertia	6.3 kg·mm^2^
Internal resistance	250.1Ω
Inductance	1.12 × 10^−3^ H
Back electromotive voltage constant	0.24 V·s/rad
Motor External Diameter	43 mm
Motor external height	67.2 mm

Bringing (Equation 14), (Equation 15), (Equation 16) into (Equation 17), we can obtain:

(Equation 18) $$P_{E}=\frac{1.5E_{m}^{2}}{R_{e}+R_{l}}$$

where $R_{e}$ is the internal resistance of the motor and $R_{l}$ is the external load resistance in the circuit.

### Test and result discussion

#### Test setup

In order to effectively evaluate the output performance of the SOR-SSS, we utilized 3D additive printing technology to fabricate a prototype and placed it on a six-degree-of-freedom vibration test bench, as depicted in Figure 4. This setup allowed us to simulate the actual ocean ranch environment. The six-degree-of-freedom vibration test bench primarily provided controlled pitching excitation at various frequencies and angles. We recorded the voltage data obtained from the experiment using a RIGOL DS1102 oscilloscope. To regulate the performance output under different loads and calculate the actual output power at the load, we prepared a resistor box model SHANE ZX99-IA. Additionally, we used capacitors, wireless temperature, and humidity sensors, and light strips along with other devices for subsequent performance testing of the SOR-SSS. Figure 4B illustrates the six different sizes of the excited-toothed rings of the PEHU cam excitation module. Figure 4C showcases the prototype of the shafting unit of the SOR-SSS system. Figure 4D displays the prototype of the PEHU, which is excited by the rotation of the red cam portion upon contact with the PEHU, thereby generating the electrical signal.

#### Optimal configuration test

To determine a suitable PEHU excitation configuration for the SOR-SSS, we conducted a configuration mode selection experiment prior to the performance testing. The goal was to identify the optimal configuration of the SOR-SSS based on the voltage performance of the PEHU under different protruding distances and varying numbers of cams in the cams. We also referred to fishery literature and performed pitching motion simulations of the floating body. Additionally, we considered the working characteristics of the six-degree-of-freedom vibration test bench. Taking into account the aforementioned factors, we established the experimental conditions as follows: a frequency range of 0.1 Hz–0.5 Hz and a maximum pitching angle of 12°. Considering that piezoelectric and electromagnetic power generation methods can provide higher voltage under the same experimental conditions, we use the piezoelectric power generation method as an element to measure the optimal configuration of the SOR-SSS.

Figures 5A and 5B show the root-mean-square (RMS) and maximum voltage performance of different numbers of rings under different numbers of rings with a protrusion distance of 3 mm and 5 mm, respectively, with a working condition of 0.1 Hz, 0.2 Hz, 0.3 Hz, 0.4 Hz, 0.5 Hz and a maximum pitch angle of 12°. We can see that under the same number of cams and working conditions, the more cams, the better its voltage performance; When the number of cams is 2 or 4, the maximum voltage value is higher than the RSM voltage value in the low-frequency environment, regardless of whether the protrusion distance is the same or not. The reason for this is that when the number of cams is small, the frequency of excitation is lower; However, when the number of cams is 8, the RMS voltage is larger than the maximum voltage, regardless of whether the protrusion distance is 3 mm or 5 mm, so when selecting a SOR-SSS configuration, we should choose a configuration with a large number of cams as much as possible. Figures 5C and 5D display the voltage performance at different frequencies for a protruding distance of 3 mm and 5 mm, respectively, with 8 cams. We can also see that with the increase of the experimental frequency, the more the number of electrical signal peaks and troughs generated by PEHU within 12 s, the more excited frequencies increase. the excitation frequency at a protrusion distance of 5 mm is larger than that at 3 mm. Additionally, Figures 5E and 5F present the corresponding 12 s voltage data in the 0.4 Hz experimental environment. Based on the above descriptions, we ultimately selected the toothed ring with a protrusion distance of 5 mm and 8 cams as the optimal configuration for the SOR-SSS, which would be used for subsequent experiments on the performance output of the SOR-SSS.

#### Device performance

In Figure 6A, the average pitching angle of the floating body at various frequencies and wave heights is depicted using the simulation software ANSYS AQWA. It can be observed that the average pitching angle is predominantly concentrated around 10°–15°. Figure 6B illustrates the pitching angle of the floating body at different frequencies. Figure 6C presents the impact of load resistance on the RMS voltage and output power of electromagnetic power generation under different pitching oscillation frequencies. The effective RMS voltage of electromagnetism can be directly measured using an oscilloscope, and the output power can be calculated using the formula $P=V_{rms}^{2}/R_{load}$, where $R_{load}$ represents the external load resistance. The figure demonstrates that the output power varies with changes in the external load. Notably, at an external load resistance of approximately 250 Ω, the output power for three operating conditions reaches its maximum value. To simplify calculations, we selected an external load resistance of 250 Ω for subsequent electromagnetic laboratory experiments to maximize the electromagnetic power. In Figure 6D, for four pitching angles, when the angle reaches 12°, the electromagnetic power reaches 4.21 mW at 0.5 Hz. Even at 6°, the electromagnetic output power at 0.5 Hz reaches 2.72 mW. In Figure 6E, this figure briefly explores the power generation performance of piezoelectric devices without specifically discussing the impact of external resistance changes on piezoelectric power generation performance. The external resistance was set to 20 kΩ as the load for the piezoelectric device. It can be observed that the piezoelectric unit exhibits turning points in four pitching angles, gradually increasing from ultra-low frequencies (0.1 Hz) to the turning point at 0.5 Hz. This suggests that the piezoelectric device performs optimally at 0.4 Hz. When the pitching angle is 12°, the maximum output power reaches 3.55 mW Figure 6F presents a comparative bar analysis of the power generation of two types of power generation devices at different frequencies (with a pitching of 12°) and the ratio of the PEHU output power to the total output power. When considering the power generated by each individual power-generating unit, the output power of the EEHU increases from 0.06 mW to 4.21 mW as the frequency increases. On the other hand, the output power of the PEHU module initially rose from 0.26 mW to 3.55 mW and then decreased to 2.81 mW. However, as the frequency increases, the PEHU’s power generation performance as a percentage of the sum of the two decreases from 81.14% to 39.99%. This observation suggests that the PEHU exhibits better stability than the EEHU at low frequencies and that the power generated by the PEHU is relatively less affected by fluctuations in the excitation frequency. At a frequency of 0.4 Hz, the SOR-SSS system attains a remarkable maximum total hybrid output power of 17.56 mW.

#### The performance analysis of practical applications

To further evaluate the performance of the SOR-SSS, we conducted experiments involving LED power supply and wireless sensor power supply, as depicted in the enlarged diagram in Figure 7A. For the LED power supply experiment, we utilized a matrix lamp with “IOT” characteristics, consisting of a total of 152 LED lamps. Prior to the charging experiment, we connected the four piezoelectric plates of the piezoelectric energy acquisition module in parallel and rectified the output through a rectifier. In the electromagnetic energy acquisition module, we directly connected the electrical signal generated by the generator to the rectifier bridge. Finally, the signals from both modules were connected to the voltage regulator module of the MT3608 model, which supplied power to the capacitor. Considering the output power performance of the SOR-SSS, we selected the experimental conditions for charging and discharging as 0.4 Hz with a pitching angle of 10°. In Figure 7B, it can be observed that the two modules are combined to charge four capacitors with different capacities. Due to the large capacity of the capacitors, the charging times for all four capacitors exceeded 90 s to reach equilibrium. Figure 7C illustrates the charging and discharging experiment. The A-B stage represents the waiting stage during which the SOR-SSS is not operational. The B-C is in the charging phase, followed by the C-D stage, which represents the first working stage of the temperature and humidity wireless sensor. The D-E stage indicates the charging stage after the first working stage, while the E-F stage represents the second working stage of the temperature and humidity sensor, which is connected to the mobile phone via an app. Finally, the F-G stage represents the second charging stage until the charging process is completed. The entire process took approximately 220-s. In Figure 7D, it can be observed that the two modules are combined to charge four capacitors with different capacities. Similar to the previous case, the charging times for all four capacitors exceeded 90 s to reach equilibrium. Figure 7E presents the power management circuit of the SOR-SSS for supplying power to wireless sensors. Due to limitations in the experimental conditions, we conducted field verification at the source of the Yangtze River in Yi bin City, Sichuan Province, China, as shown in Figure 7F. The floating bodys were arranged in a circular pattern, and the pitching motion generated by the floating bodys was not very strong when subjected to the natural wave conditions of the Yangtze River. However, when a cargo ship passed by, the wave generated by its passage provided a suitable simulation of real fishery wave environments, gradually enhancing the pitching motion of the floating bodys. The observed angles were generally consistent with the simulated angles.

The SOR-SSS effective volume is π×140^2^ mm^3^. Based on the above analysis of the experimental results, the maximum average power density can be found as 4.76 W m ^−3^ Hz ^−1^, It can effectively solve the problem of sustainable power supply for wireless sensor network nodes, and at the same time, in terms of ultra-low-frequency wave energy harvesting. There also exist some harvesters with excellent performance. Feng et al.^35^ proposed a hybrid wave energy collector that can also collect ultra-low-frequency wave energy, and its power density is 2.30 W m ^−3^ Hz ^−1^. The energy harvester proposed by Tian et al.^43^ that combines the three modes of power generation has excellent performance in broadening the bandwidth of the capture frequency, and its power output density can reach 1.78 W m ^−3^ Hz ^−1^. This article also includes the power consumption data of various common sensors deployed in the ocean ranch environment. The specific sensor models and their power consumption are presented in Table 3. Assuming that the SOR-SSS operates effectively for 16 h per day, and there are three SOR-SSS units arranged around the circumference of each ocean net cage, the power generated per net cage per day can reach up to 3034.36 J. On average, each type of sensor around the net cages is equipped with one unit. Considering this, the average power consumption of wireless sensors arranged around a net cage is 1693.44 J per day, which is sufficient to support the power requirements of the surrounding sensors.

**Table 3:** Power consumption table for common ocean sensors

Sensor name	Type	Power consumption
Dissolved Oxygen Sensor	KDS-25B	10 mW
Miniature Pressure Sensors	LFC-07	9 mW
Temperature and Humidity Sensors	DHT11	0.6 mW

#### Smart ocean ranch monitoring based on LSTM

Incorporating a machine learning component into the original single system enables the identification of the operating status of critical parts or types of faults, and ultimately improves the overall stability of the system.^53^ In the context of SOR-SSS implementation in ocean ranches, the detection of abnormal conditions may be delayed due to prolonged exposure to external factors such as waves and currents inherent in the ocean environment. Additionally, it is challenging for conventional detection devices to operate effectively in such an environment for an extended period. To address this issue, we employ the LSTM model of the neural network algorithm to train and process the signals collected by the SOR-SSS. This enables the categorization of different abnormal conditions, thereby facilitating the detection of anomalies in the current device and its surrounding environment.

LSTM, a recurrent neural network, is widely utilized in analyzing sequence data and predicting time series. It is particularly effective in capturing long-term dependencies.^54^^,^^55^ In this experiment, we obtained multiple sets of voltage signals captured during both normal and non-normal operating conditions of the SOR-SSS. The types of signals are illustrated in Figure 8A. The specific architecture of the LSTM model is depicted in Figure 8B, and the subsequent data preprocessing and training process are presented in Figure 8C. These steps include: slicing the signals using the sliding window method to enhance data features and improve training generalization; labeling different categories of voltage signals; and training the LSTM model by dividing the dataset into a 7:3 ratio for the training and test sets, ultimately obtaining the classification detection results. Furthermore, to detect abnormal environmental conditions in the ocean, we collected additional datasets under both normal and abnormal wave conditions. These datasets were incorporated into the same training process to obtain results for the detection of abnormal environmental conditions.

The accuracy and loss value curves during the training iterations of the network model we used are shown later in discussion. The classification task for waves of different frequencies in the environment is illustrated in Figure 8D, where the training accuracy is able to reach up to 99.72%. Similarly, for the task of detecting normal anomalies in device conditions, the training accuracy reaches a commendable 99.80%. Moreover, the training loss for both tasks can be reduced to a very low level. Furthermore, the testing results of the model are presented in the confusion matrix depicted in Figure 8E for the frequency classification task and Figure 8F for the condition classification task. These results indicate that the testing accuracy for the frequency classification task exceeds 95%, while the testing accuracy for the condition classification task is above 97%. The simulation results collectively demonstrate the successful application of the self-monitoring system in our device. In summary, the signal analysis of SOR-SSS using the LSTM model offers a data-driven approach to monitor and evaluate the operational status of power generation equipment and the overall health of the ocean environment in ocean ranch applications. This enables the SOR-SSS to detect anomalies at an early stage, enabling timely intervention and maintenance to ensure sustainable and efficient operation of ocean ranch.

#### Application outlook

**Figure 9:**
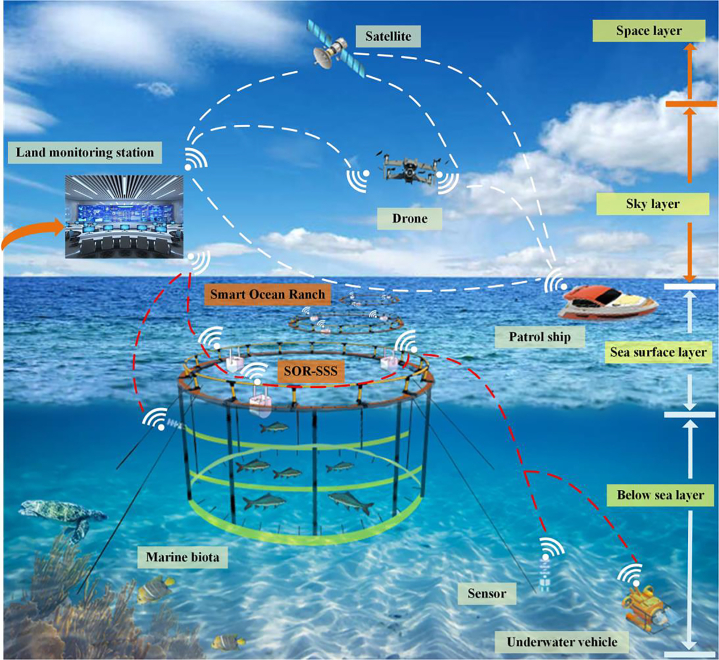
Application potential of SOR-SSS in offshore smart ocean ranch

In ocean ranch located closer to the shore, there are some difficulties in the transmission and maintenance of electricity, resulting in inconveniences for the operation of smart ocean ranch. To address this issue, we propose the development of the SOR-SSS ring ocean ranch array arrangement. This arrangement effectively collects wave energy from the surrounding ocean ranch environment, converts it into required electricity in a timely manner, and establishes a real-time prediction and monitoring system. This solution effectively resolves the difficulties and high costs associated with power supply changes for the nodes of ocean ranch sensors. As depicted in Figure 9, the application of the proposed SOR-SSS in the smart ocean ranch environment demonstrates its potential as a power source for smart ocean ranch. Additionally, this hybrid energy harvesting system contributes to reducing carbon emissions, making a meaningful contribution to environmental sustainability.

### Conclusions

In this article, we present a self-powered and self-monitoring wave energy harvester designed for smart ocean ranch applications. Taking into consideration the unique characteristics of the environment, this system primarily focuses on harnessing the energy generated by pitching motion. The SOR-SSS achieves self-powering through an energy input module, energy harvesting module, and energy conversion module. Additionally, it incorporates self-monitoring capabilities utilizing the LSTM algorithm for processing basic experimental data. To validate the proposed SOR-SSS, we first establish a theoretical model. Subsequently, we employ AQWA hydrodynamic simulation software to simulate the pitching motion of the floating body where the SOR-SSS system is installed. Wave simulation experiments are then conducted using a six-degree-of-freedom vibration test bench capable of replicating wave conditions. Due to experimental constraints, we also deploy the floating body, which carries the SOR-SSS system, in the Yangtze River source, utilizing waves generated by passing ships to simulate the desired wave motion. Experimental results from the six-degree-of-freedom vibration test bench demonstrate that the EEHU achieves a maximum total power output of 4.21 mW under an external load of 250 Ω. At an experimental frequency of 0.5 Hz and a pitching angle of 12°, the system reaches its maximum total power output. Similarly, the PEHU achieves a maximum output power of 3.55 mW with an external load of 20 kΩ at an operating frequency of 0.4 Hz. The maximum theoretical output power of the system is 17.56 mW at 0.4 Hz. Furthermore, we employ the LSTM algorithm to predict the basic data of the PEHU obtained from experiments. This enables real-time monitoring and prediction of wave frequency and equipment status, achieving accuracy rates of 99.72% and 99.80%, respectively. Additionally, we verify the reliability of the system through experiments involving LED pointing lights, charging and discharging capacitors, and powering wireless sensors with commercial value. Overall, the SOR-SSS system exhibits significant potential for application and contributes to the development of a comprehensive and modernized industry chain system for smart ocean ranch.

### Limitations of the study

This question proposes a self-powering and self-monitoring system applied to the smart ocean ranch, which collects ultra-low-frequency wave energy from the ocean ranch for self-powering low-power devices on the one hand, and supervises the environmental working conditions by utilizing deep learning on the other hand. However, the proposed system still needs further optimization analysis. The limitations of this study are summarized as follows.

(1) In this study, we assumed that the floating body is fixed to the net box by hinges and only considered the working condition of SOR-SSS when the floating body moves in a pitching motion. However, in this case, there is also a small heaving motion of the floating body, and in future studies, we will analyze the combined motion of the floating body considering this small amount of motion into account.
(2) It is also worth noting that because of the limitations of our experimental conditions, we were unable to conduct field experiments in this study, and in order to better derive the performance of the SOR-SSS, we will test it on actual ocean nets in future studies.
(3) The clean energy around the ocean ranch environment includes not only wave energy but also solar energy and wind energy, and so forth. In the subsequent research, we will optimize the structural layout of SOR-SSS and design more kinds of capture units to achieve the collection of more energy.

## STAR★Methods

### Key resources table

**Table:** 

REAGENT or RESOURCE	SOURCE	IDENTIFIER
**Software and algorithms**		

Workbench 2022 R1	Workbench	https://www.ansys.com/zh-cn/products/ansys-workbench
Origin 2018	Originlab	https://www.originlab.com/
Microsoft Visio 2016	Microsoft	https://www.microsoft.com/zh-cn/microsoft-365/visio/flowchart-software

**Other**		

DS1102Z-E digital oscilloscope	RIGOL	https://rigol.com

### Resource availability

#### Lead contact

Further information and requests for resources and reagents should be directed to and will be fulfilled by the lead contact Zutao Zhang (zzt@swjtu.edu.cn).

#### Materials availability

This study did not generate new unique reagents.

#### Data and code availability

• All data reported in this paper will be shared by the lead contact upon reasonable request.
• This paper does not report original code.
• Any additional information required to reanalyze the data reported in this paper is available from the lead contact upon request.

### Method details

All methods can be found in the main text. Please check the System Designed to Work section for detailed information on the system’s components and working principles. Please check the System Dynamic Analysis section for complete information on the system’s state of motion. Please check the System Experimental Setup and Performance Analysis section for detailed information on the experimental environment and system output performance. Microsoft Visio 2016 was used to generate the visual images in the manuscript. Origin 2018 was used to process the experimental data and generate visual images in the manuscript. A demo video is provided to present the entire article more vividly.

### Quantification and statistical analysis

Workbench 2022 R1 was used for hydrodynamic simulation of floating body. Microsoft Visio 2019 was used to generate visual images in the manuscript. Origin 2018 was used to process the experimental data and generate visual images in the manuscript. Voltage signals were captured by digital oscilloscope (RIGOL DS1102).

## Supplemental information

Video S1. Demo video, related to STAR Methods
